# Maternal Disorders Associated with Morbidity and Mortality in a Metropolis of Kazakhstan

**DOI:** 10.3390/clinpract15060108

**Published:** 2025-06-06

**Authors:** Aigerim Turekulova, Nurzhamal Dzhardemaliyeva, Alibek Mereke, Mukhtar Kulimbet

**Affiliations:** 1 School of General Medicine, Asfendiyarov Kazakh National Medical University, 050012 Almaty, Kazakhstan; a.turekulova@kaznmu.kz; 2 International Faculty, Asfendiyarov Kazakh National Medical University, 050012 Almaty, Kazakhstan; dzhardemalieva.n@kaznmu.kz; 3 Faculty of Medicine and Health Care, Al-Farabi Kazakh National University, 050040 Almaty, Kazakhstan; alm312@pitt.edu; 4 Science Department, Asfendiyarov Kazakh National Medical University, 050012 Almaty, Kazakhstan

**Keywords:** Disability-Adjusted Life Year, maternal disorders, hypertensive disorders of pregnancy, pre-eclampsia, eclampsia, Kazakhstan

## Abstract

**Background/Objectives:** Hypertensive disorders of pregnancy, including pre-eclampsia and eclampsia, are leading causes of maternal morbidity and mortality worldwide and in Kazakhstan. This study aims to assess the burden of hypertensive disorders of pregnancy and main maternal disorders in Almaty, Kazakhstan, using the disability adjusted life years (DALY) methodology. **Methods**: We conducted a retrospective analysis of women aged 18 and above in Almaty, Kazakhstan, from 2018 to 2020. The medical claim data were retrieved from the Almaty city branch of the Republican Center for Electronic Health Care. Incidence-based DALY were calculated. **Results**: The total DALY increased for severe pre-eclampsia from 109.3 in 2018 to 187.2 in 2020 per 100,000 population and eclampsia from 3.1 in 2018 to 159.3 in 2020 per 100,000 population. Also, the 25–29 years age group had the largest increase in percent change (5.8) in the total DALY for hypertensive disorders of pregnancy. In addition, the 20–24 years age groups had the largest increase in percent change in the total DALY for severe pre-eclampsia (25.8) and eclampsia (80.5). **Conclusions**: Our findings highlight an increase in the burden of maternal disorders, especially for severe pre-eclampsia and eclampsia, in Almaty, Kazakhstan, from 2018 to 2020. Younger women bear a significant share of this burden, compounded by the pandemic’s impact on healthcare services.

## 1. Introduction

Maternal health, which refers to the health of women during pregnancy, childbirth, and the postpartum period [1], is a critical aspect of public health and a significant indicator of a nation’s overall health and development worldwide. Maternal health is a specific focus of the United Nations Sustainable Development Goals (SDG), where target 3.1 aims to reduce the global maternal mortality ratio (MMR) [2] to less than 70 per 100,000 live births by 2030 [3], and Kazakhstan’s one of the goals to lower its MMR align with SDG targets. Thus, to achieve this target, it is essential to identify various maternal disorders that significantly impact maternal health and contribute to maternal mortality.

Over the last two decades, Kazakhstan has implemented several national health programs [4,5,6], aimed at improving maternal health and to enhance the accessibility and quality of maternal care services. These programs align with the World Health Organization’s (WHO) definition of reproductive and maternal health services [7]. For instance, the State Program for Healthcare Development for 2020–2025 “Healthy Nation” [6] continues to improve maternal health by increasing coverage for comprehensive reproductive health services, accessing advanced maternal care, and the integration of modern medical technologies to reduce maternal morbidity and mortality in Kazakhstan. As a result of the national health programs, Kazakhstan was one of the 10 countries with the largest percentage reduction in the MMR between 2000 and 2020 in the world, with the overall percent change (reduction) of 76.3% [2].

It is important to understand the pattern of maternal disorders, which constitute a significant global health burden and are leading causes of death and disability among women of reproductive age [8,9,10]. Hypertensive disorders of pregnancy (HDP), including pre-eclampsia and eclampsia, along with maternal hemorrhage, are primary causes of maternal complications worldwide, especially in low- and middle-income countries [8,9,10]. The incidence of HDP increased globally from 17.5 million to 18.1 million between 2010 and 2021 [11]. The global age-standardized prevalence rate of HDP was 44.4 per 100,000 population in 2021 [11]. The burden remains high globally, particularly in areas with lower sociodemographic and human development indices [12,13]. In Kazakhstan, the epidemiological landscape of maternal disorders mirrors global trends, particularly HDP along with maternal hemorrhage being the major contributors of maternal mortality and morbidity [14,15,16].

To better understand the responsiveness of the maternal health services to the existing and emerging maternal health challenges, especially during health emergencies and pandemics, we employed the Disability-Adjusted Life Years (DALY) methodology [17,18], a comprehensive approach for quantifying the burden of disease that measures the combined impact of morbidity and mortality, including severity of disease, thereby taking into account the true health lost [19]. As a result, we could see the specific contours of maternal health challenges in Kazakhstan, offering a foundation for targeted interventions and policy adjustments.

The aim of the study was to assess the burden of HDP and main maternal disorders in the Almaty metropolis of Kazakhstan, using the DALY methodology. This study provides a detailed understanding of the current state of maternal disorders in Almaty, Kazakhstan, and contributes to the development of targeted strategies to improve maternal health outcomes.

## 2. Materials and Methods

### 2.1. Study Design

The study population consisted of adult women patients aged 18 and above years in both inpatient and outpatient clinical settings for treatment regarding maternal disorders during 2018–2020 in Almaty, Kazakhstan. Kazakhstan is a middle-income country in Central Asia. Almaty is the largest megalopolis of Kazakhstan with 2 million population, located in the southeast region of the country.

### 2.2. Data Sources

The medical claim data were retrieved from the Almaty city branch of the Republican Center for Electronic Health Care (RCEH), which promotes the development of the healthcare system in the country by strengthening the information infrastructure of the healthcare system and medical statistics (https://rcez.kz/aboutcompany, accessed on: 6 August 2021).

Aggregated census data with a 5-year interval on women starting from the age of 15 years and above for the corresponding period were obtained from the Bureau of National Statistics Agency for Strategic Planning and Reforms of the Republic of Kazakhstan (BNSA). The BNSA is a government entity that collects, analyzes, and disseminates national statistics related to various socioeconomic and demographic aspects of the country, informing policy decisions, strategic planning, and reforms across multiple sectors of Kazakhstan (https://stat.gov.kz/en/about/description/, accessed on: 10 August 2021).

### 2.3. Patient Registration Processes in Kazakhstan

A registration of pregnancy in Kazakhstan is done after confirmation. Once the pregnancy is confirmed, the woman should be registered by 12 gestational weeks as per national regulation as a pregnant patient in the medical information system (MIS). This registration occurs at her local outpatient clinic. All pregnant patients are linked to antenatal care for pregnancy management and complication prevention. In Kazakhstan, citizens are granted medical care, including maternal health services, as part of the guaranteed free health package. In cases where pregnant women develop maternal disorders or when complications arise, inpatient care may be necessary. Kazakhstan’s healthcare system provides specialized care for such cases, including hospitalization, multidisciplinary care, emergency care, and postnatal follow-up.

Each citizen of Kazakhstan has a unique code called the Individual Identification Number (IIN), used across various administrative processes, including healthcare services. The IIN is linked with a patient’s medical records. Health data generation starts with a doctor’s visit or hospital admission when a patient is registered in a medical information system (MIS). During registration, demographic information such as name, date of birth, address, contact details, and IIN is collected. An Electronic Medical Record (EMR) containing comprehensive clinical information is created and used for patient management. Discharge data from the EMR is transferred to a centralized health information system called the Electronic Register for Inpatient Patients (ERIP). The ERIP automates data collection on treated cases in inpatient settings based on hospital statistics, discharge, and claim data for reimbursement from the republican budget, and provides statistical and analytical reporting on hospital activities and patient structure (https://rcez.kz/informationsystems, accessed on: 6 August 2021). A patient and the healthcare system interaction generate health data recorded in EMRs, including name, IIN, unique identifier code, diagnosis based on International Classification of Diseases-10 (ICD-10), healthcare providers, services rendered based on International Classification of Diseases-9 (ICD-9), and outcomes. Patient registration and data generation in Kazakhstan follow national regulations for data protection and privacy.

### 2.4. Patient Identification and Definitions

A total of 232,308 records of women registered with maternal disorders were obtained. Out of all, 50,853 records were selected based on Global Burden of Diseases (GBD) case definitions [20] and ICD-10 codes (https://icd.who.int/browse10/2019/en, accessed on 6 August 2021) for hypertensive disorders of pregnancy including pre-existing hypertension complicating pregnancy (O10–O13, O16), severe pre-eclampsia (O14.1), eclampsia (O15), maternal hemorrhage (O20, O44–O46, O67, O72), abortion (O00–O08), obstructed labor (O64–66) and maternal sepsis (O85, O75.3). These case definitions are presented in Table 1.

**Figure 1:**
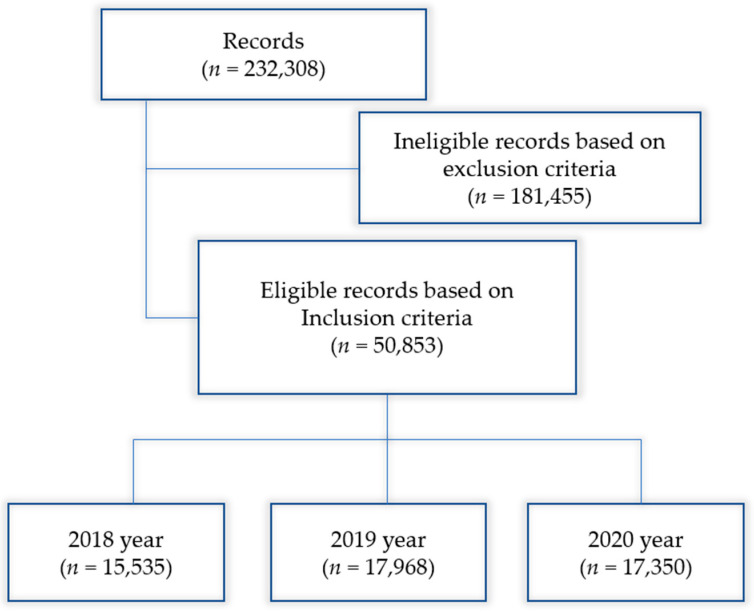
Data identification flow chart. Initial data included 232,308 records. Based on inclusion and exclusion criteria we ended up with 50,853 records for the 2018–2020 period.

Among 50,853 records, there were 15,535 records in 2018, 17,968 records in 2019, and 17,350 records in 2020, respectively (Figure 1).

### 2.5. DALY Calculation

To measure maternal disorders burden, we estimated incidence-based DALYs, which include years of life lost (YLL) and years lived with disability (YLD). The basic formula for DALY looks like this [21]:

DALY = YLL + YLD = N × L1 + I × DW × L2

YLL is calculated as the number of deaths (N) × the standard life expectancy at age of death (L1). YLD is the number of new cases of a disease (I) × a disability weight (DW) × the average time a person lives with the disease before remission or death (L2).

YLL was calculated using cause of death data from the RCEH. Life expectancy was based on the general population’s life expectancy by age, using life tables of the BNSA for 2018, 2019, 2020.

The YLD was calculated by using the incidence rate, age at onset, disease duration, and disability weight. Estimates of incidence were based on the prevalence of cases from the claim data. Disability weights were taken from health states and disability weights for each of the nonfatal maternal disorders [22].

In addition, we used standard population as well as age standardized rates, which provides a rate that reflects the number of YLD, YLL, or DALY per 100,000 population.

All analyses were performed using SAS OnDemand for Academics (release 3.81, Carry, NC, USA) and Microsoft Excel (version 18.2311.1071.0 Microsoft Corporation).

### 2.6. Ethics

The study was conducted in accordance with the Declaration of Helsinki, and approved by the Institutional Review Board of Asfendiyarov Kazakh National Medical University (protocol code 873), date of approval 25 March 2020. Informed consent was waived by the board due to the nature of the study, which involved the secondary analysis of deidentified data.

## 3. Results

The results provided represent the incidence-based DALYs per 100,000 population for maternal disorders categorized by age groups, including HDP, severe pre-eclampsia, eclampsia, maternal hemorrhage, abortion, obstructed labor, and maternal sepsis for the years of 2018–2020 separately. Please refer to the supplementary material for a detailed information of the incidence-based DALYs per 100,000 population for maternal disorders categorized by age groups for the years 2018–2020 separately (Tables S1–S3). The DALYs of maternal disorders were highest in the 20–24 years age groups and lowest in the 45–55 years age groups.

**Figure 2:**
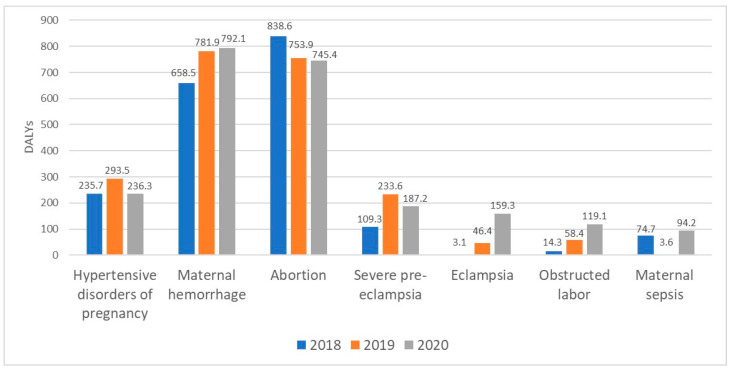
Disability-Adjusted Life Years in Almaty from 2018 to 2020 by Maternal Disorder (per 100,000 population).

Figure 2 displays the DALYs for various maternal disorders from 2018 to 2020. The total DALY for HDP was 236.3 per 100,000 population in 2020. The total DALY for severe pre-eclampsia and eclampsia was increased from 109.3 in 2018 to 187.2 and from 3.1 in 2018 to 159.3 per 100,000 population in 2020 accordingly. The total DALY for maternal hemorrhage increased from 658.5 per 100,000 population in 2018 to 792.1 per 100,000 population in 2020.

**Figure 3:**
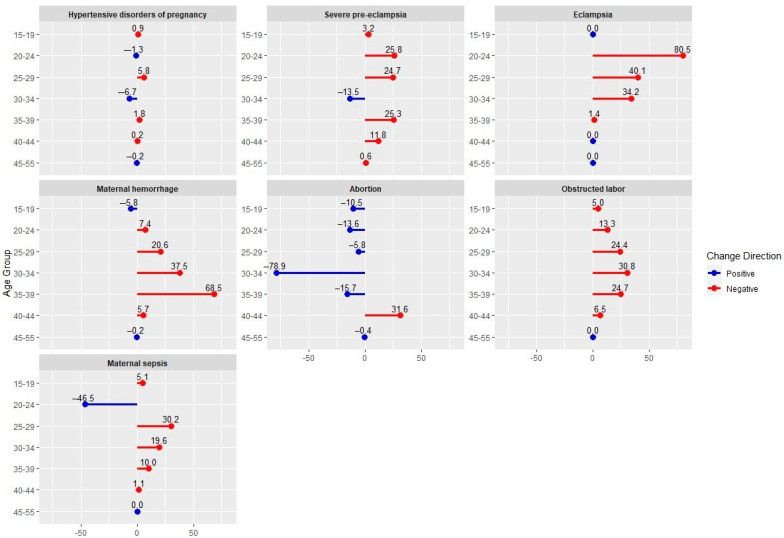
Change in Disability-Adjusted Life Years in Almaty from 2018 to 2020 by age group and Maternal Disorder (per 100,000 population).

Figure 3 shows the change in DALY per 100,000 population from 2018 to 2020 by age group and maternal disorders. There was a slight negative change in the total DALY per 100,000 population for HDP in most of the age groups from 2018 to 2020 with the 25–29 age group showing the largest increase of 5.8 DALY per 100,000 population. Furthermore, there was a negative change in the total DALY per 100,000 population almost in all age groups for severe pre-eclampsia and eclampsia from 2018 to 2020. The age groups 20–29 showed the highest negative change of 25.8 in the total DALY per 100,000 population for severe pre-eclampsia. Also, age groups 20–24 showed the highest negative change of 80.5 in the total DALY per 100,000 population for eclampsia. Please refer to the supplementary material for a detailed description of change in Disability-Adjusted Life Years in Almaty from 2018 to 2020 by maternal disorder (Figures S1 and S2).

## 4. Discussion

This study is the first to assess the burden of hypertensive disorders of pregnancy (HDP) and main maternal disorders (MD) in the megapolis Kazakhstan using the DALY methodology. Our findings highlight significant trends in maternal health from 2018 to 2020, particularly concerning severe pre-eclampsia and eclampsia. We found that the total DALY for HDP increased between 2018 to 2020, particularly among pregnant women with severe pre-eclampsia and eclampsia 187.2 and 159.3 per 100,000 population accordingly. In addition, there was an increase in the total DALY for HDP (0.6) along with severe pre-eclampsia (77.9) and eclampsia (156.1) per 100,000 population from 2018 to 2020. Furthermore, the DALYs of HDP showed the largest increase in percent change in the 25–29 years age group. Also, the DALYs of severe pre-eclampsia and eclampsia had the largest increase in percent change in the 20–24 years age group. While HDP did not constitute the primary cause of maternal disorders in Almaty, Kazakhstan, there was a notable correlation between their DALY and the volume of abortions. These insights underscore the pressing need for focused strategies to tackle maternal health challenges in Kazakhstan.

HDP, including pre-eclampsia and eclampsia, have been identified as significant contributors to the overall DALYs across various age groups. Our findings indicated that younger women in the 20–24 years and 25–29 years age groups experience a burden from eclampsia and severe pre-eclampsia. Several factors likely contributed to this trend. For example, the COVID-19 pandemic disrupted healthcare systems globally, leading to reduced access to antenatal care [23,24]. Many pregnant women faced challenges in obtaining timely and regular antenatal check-ups, which are crucial for monitoring and managing conditions like HDP to prevent pre-eclampsia and eclampsia. Our data demonstrated that limited access to healthcare services during the pandemic likely contributed to the increased severity and late detection of these conditions. This aligns with studies conducted in Kazakhstan [25], where similar disruptions in healthcare services were reported, and as well as in other studies worldwide [26]. In addition, during the pandemic, many healthcare facilities had to reallocate resources and personnel to manage COVID-19 cases [27]. This led to a reduction in routine healthcare services, including antenatal care.

Another important finding from our study was the observed correlation with decrease in the frequency of abortions and increase of HDP and other maternal disorders, particularly notable in the year 2020. This could be partly explained by limited access to family planning and primary care services, contraceptive methods, and abortion due to the COVID-19 pandemic and disrupted healthcare system. The pandemic led to widespread restrictions and lockdowns, which disrupted many healthcare services, including sexual and reproductive health services [28]. Outpatients and hospitals faced significant challenges in maintaining routine operations, prioritizing COVID-19 cases, and implementing safety protocols, which resulted in reduced availability and access to abortion services [29,30].

The “Densaulyk” state healthcare development program (2016–2019) in Kazakhstan played a crucial role in shaping maternal health outcomes [5]. As a result, improved screening and diagnostics at antenatal care with digitization of electronic medical information systems could have had impacts on the burden of HDP. For example, advancements in antenatal screening and diagnostic techniques have enabled earlier and more accurate identification of HDP and other maternal disorders, resulting in more cases being captured and reported [31]. Implementation of electronic medical records have improved data collection, monitoring, and reporting [32,33]. We could also observe a similar pattern in our study where more cases in maternal disorders had been captured and reported starting from 2019. This coincides with a mass use of electronic medical health information system in Kazakhstan that was launched in November 2018.

### 4.1. Public Health Relevance

We suggest potential interventions and policy changes to mitigate the burden of HDP, focusing on prevention, early detection, effective management, and continuous improvement of maternal health services. First, enhanced antenatal care programs could magnify the early detection and identify women at high risk. For example, self-monitoring of blood pressure during pregnancy could be effective for the early diagnosis of gestational and masked hypertension [34,35]. Also, conducting self-testing of urine for protein among pregnant women at risk to identify signs of preeclampsia [36,37]. Second, raising awareness of HDP is critical to improving early detection and maternal health services. This requires a multifaceted approach involving education campaigns, healthcare provider training, patient engagement, and policy advocacy is essential. Finally, non-governmental organizations (NGOs) can play a crucial role in advocating for stronger maternity protection policies. NGOs can raise awareness about the importance of maternity protection among policymakers and the general public. Thus, NGOs can advocate for policy changes, provide education and training, deliver essential healthcare services, and support economic empowerment.

### 4.2. Limitations of the Study

The study, while offering valuable insights, is subject to few limitations. The inability to perform deduplication due to the absence of unique patient identifiers may have led to potential overestimation or underestimation of the DALYs. The DALY calculation for the age group of 15 to 19 years may be underestimated because the numerator includes only women aged 18 to 19 years due to data restrictions, while the denominator accounts for all women aged 15 to 19. The lack of information on whether the pregnancy was the first or subsequent introduces another layer of uncertainty, as the risk profiles and outcomes can vary significantly between first and later pregnancies. Furthermore, the reliance on ICD-10 codes without detailed descriptions for case coding of YLL data may have limited the granularity and specificity of the data captured, particularly concerning maternal deaths. Cultural characteristics of different ethnic groups, which might influence maternal health behaviors and access to care, were not accounted for in this study. potentially overlooking important contextual factors affecting maternal health outcomes.

### 4.3. Recommendations for Future Research

To better understand HDP, including pre-eclampsia and eclampsia, further research is needed in prevention strategies and diagnostics and treatment. Studies should explore ways to enhance the role of family planning services in providing education and improving access to contraceptives. Additionally, research should focus on optimizing primary healthcare services for the early diagnosis and continuous monitoring of pre-existing conditions that may complicate pregnancy. Future efforts should evaluate the effectiveness of self-monitoring strategies, such as home blood pressure recording, for the early detection of gestational hypertension, white coat hypertension, and masked hypertension. Investigating the feasibility and impact of urine self-testing for proteinuria among high-risk pregnant women can provide valuable insights into early preeclampsia detection. Furthermore, research should address interventions to improve patient adherence to antenatal visits and the implementation of preventive and therapeutic measures based on evidence-based medicine.

## 5. Conclusions

This study is the first to evaluate maternal health services in Almaty, Kazakhstan, from 2018 to 2020 using the DALY methodology. It highlights a significant increase in the burden of HDP, particularly severe pre-eclampsia and eclampsia, during this period. The study underscores the impact of the COVID-19 pandemic on healthcare access, contributing to the severity and late detection of these conditions. Notably, younger women, especially in the 20–24 and 25–29 age groups, experienced a considerable burden. The findings also suggest that improved screening, diagnostics, and digitization of medical records have led to better identification and reporting of HDP. Additionally, the study observed a decrease in abortion rates in 2020, likely due to the pandemic-related disruptions in sexual and reproductive health services. These insights emphasize the urgent need for targeted strategies to address maternal health challenges in Kazakhstan.

## Figures and Tables

**Table 1:** Categories of causes and their operational definitions.

Maternal Disorders	ICD-10 Codes	Hospitalization
Hypertensive disorders of pregnancy	O10, O11, O12, O13, O16	1 ^a^
Severe pre-eclampsia	O14.1	1 ^a^
Eclampsia	O15	1 ^a^
Maternal hemorrhage	O20, O44, O45, O46, O67, O72	1 ^a^
Abortion	O00, O01, O02, O03, O04, O05, O06, O07, O08	1 ^a^
Obstructed labor	O64, O65, O66	1 ^a^
Maternal sepsis	O85, O75.3	1 ^a^

^a^ frequency of hospitalization.

## Data Availability

The data presented in this study are available on reasonable request from the corresponding author.

## Supplementary Materials

The following supporting information can be downloaded at: https://www.mdpi.com/article/10.3390/clinpract15060108/s1, Figure S1: Change in Disability-Adjusted Life Years in Almaty from 2018 to 2020 by Maternal Disorder (per 100,000 population); Figure S2: Change in Disability-Adjusted Life Years in Almaty from 2018 to 2020 by Maternal Disorder (per 100,000 population); Table S1: Incidence-based DALYs for maternal disorders by age per 100,000 in 2018; Table S2: Incidence-based DALYs for maternal disorders by age per 100,000 in 2019; Table S3: Incidence-based DALYs for maternal disorders by age per 100,000 in 2020.

## Abbreviations

The following abbreviations are used in this manuscript:

HDP	Hypertensive disorders of pregnancy
GBD	Global Burden of Diseases
DALY	Disability adjusted life years
YLL	Years of life lost
YLD	Years lived with disability
DW	Disability weight
ICD-10	International Classification of Diseases-10
ICD-9	International Classification of Diseases-9
MMR	Maternal mortality ratio
WHO	World Health Organization
SDG	Sustainable Development Goals
BNSA	Bureau of National Statistics Agency
RCEH	Republican Center for Electronic Health Care
MIS	Medical information system
