# Supplementary material for: Maternal Disorders Associated with Morbidity and Mortality in a Metropolis of Kazakhstan
Source: Clin Pract. 2025 Jun 6;15(6):108. doi: 10.3390/clinpract15060108 (PMC12191608; doi:10.3390/clinpract15060108)
Supplement: Supplementary file 1 [file clinpract-15-00108-s001.zip › clinpract-3626115-supplementary.pdf]

As shown in Table S1, in 2018, the total DALY of maternal disorders was 1934.2 per 100,000 population. HDP (235.7 per 100,000) along with severe pre-eclampsia (109.3 per 100,000), eclampsia (3.1 per 100,000) had a moderate impact on the overall disability burden. Women aged 20–24 years had the highest DALY in HDP (66.5 per 100,000), severe pre-eclampsia (20.3 per 100,000) and eclampsia (1.6 per 100,000).

Table S1. Incidence-based DALYs for maternal disorders by age per 100,000 in 2018.

Age groups, year	HDP	Severe pre-eclampsia	Eclam psia	MH	Abor tion	OL	Maternal sepsis	Total
15-19*	14.0	6.6	-	52.4	37.4	1.5	-	111.9
20-24	66.5	20.3	1.6	220.0	147.7	4.2	74.4	534.6
25-29	51.0	12.7	1.0	154.5	149.1	3.6	-	371.9
30-34	52.3	50.3	0.6	129.0	232.1	1.6	0.1	466.0
35-39	36.2	12.1	-	76.0	170.2	3.4	0.2	298.1
40-44	14.8	6.7	-	25.4	97.5	-	-	144.5
45-55	0.8	0.6	-	1.2	4.5	-	-	7.2
All Ages	235.7	109.3	3.1	658.50	838.6	14.3	74.7	1934.2

* denominator includes women aged 15 to 19 years, but numerator includes women aged 18 to 19 years; HDP - Hypertensive disorders of pregnancy; MH - Maternal hemorrhage; OL - Obstructed labor;.

As shown in Table S2, in 2019, the total DALY of maternal disorders was 2171.4 per 100,000 population. Among maternal disorders, HDP (293.5 per 100,000) along with severe pre-eclampsia (233.6 per 100,000), eclampsia (46.41 per 100,000) had a moderate impact on the overall disability burden. Women aged 20–24 years had the highest DALY in HDP (83.6 per 100,000), severe pre-eclampsia in age group 25-29 (69.7 per 100,000) and eclampsia in age group 30-34 (40.0 per 100,000).

Table S2. Incidence-based DALYs for maternal disorders by age per 100,000 in 2019.

Age groups, year	HDP	Severe pre-eclampsia	Eclam psia	MH	Abor tion	OL	Maternal sepsis	Total
15-19*	16.8	6.6	-	68.3	38.6	1.5	0.3	132.1
20-24	83.6	41.0	4.2	245.2	142.8	5.9	1.7	524.2
25-29	66.8	69.7	1.5	185.6	144.2	11.4	1.0	480.2
30-34	60.9	28.9	40.0	144.5	174.4	19.0	0.5	468.2
35-39	46.1	73.7	0.8	100.3	161.6	17.3	0.2	399.8
40-44	18.6	12.7	-	36.8	87.9	3.4	-	159.4
45-55	0.7	0.9	-	1.3	4.4	-	-	7.4
All Ages	293.5	233.6	46.4	781.9	753.9	58.4	3.6	2171.4

* denominator includes women aged 15 to 19 years, but numerator includes women aged 18 to 19 years; HDP - Hypertensive disorders of pregnancy; MH - Maternal hemorrhage; OL - Obstructed labor;.

As shown in Table S3, in 2020, the total DALY of maternal disorders was 2333.6 per 100,000 population. Among maternal disorders, HDP (236.3 per 100,000) along with severe pre-eclampsia (187.2 per 100,000), eclampsia (159.3 per 100,000) had a moderate impact on the overall disability burden. Women aged 20–24 years had the highest DALY in HDP (65.2 per 100,000) along with severe pre-eclampsia (46.1 per 100,000), and eclampsia (82.0 per 100,000).

Table S3. Incidence-based DALYs for maternal disorders by age per 100,000 in 2020.

Age groups, year	HDP	Severe pre-eclampsia	Eclam psia	MH	Abor tion	OL	Maternal sepsis	Total
15-19*	14.9	9.8	0	46.6	27.0	6.6	5.1	109.9

20-24	65.2	46.1	82.0	227.4	134.1	17.5	27.8	600.1
25-29	56.8	37.4	41.1	175.1	143.4	28.0	30.2	512.0
30-34	45.7	36.8	34.8	166.5	153.2	32.4	19.8	489.1
35-39	38.0	37.4	1.4	144.5	154.5	28.1	10.2	414.0
40-44	15.0	18.5	-	31.1	129.2	6.5	1.2	201.5
45-55	0.7	1.2	-	1.0	4.1	-	-	7.0
All Ages	236.3	187.2	159.3	792.1	745.4	119.1	94.2	2333.6

* denominator includes women aged 15 to 19 years, but numerator includes women aged 18 to 19 years; HDP - Hypertensive disorders of pregnancy; MH - Maternal hemorrhage; OL - Obstructed labor;.

As shown in Figure S1, the total DALY for HDP almost remained the same with a slight increase from 235.7 per 100,000 population in 2018 to 236.3 per 100,000 population in 2020. Furthermore, there was a substantial increase in the total DALY for severe pre-eclampsia from 109.3 per 100,000 population in 2018 to 187.2 per 100,000 population in 2020. Also, there was a marked increase in the total DALY for eclampsia from 3.1 per 100,000 population in 2018 to 159.3 per 100,000 population in 2020. The total DALY for abortion was decreased from 838.6 per 100,000 population in 2018 to 745.4 per 100,000 population in 2020.

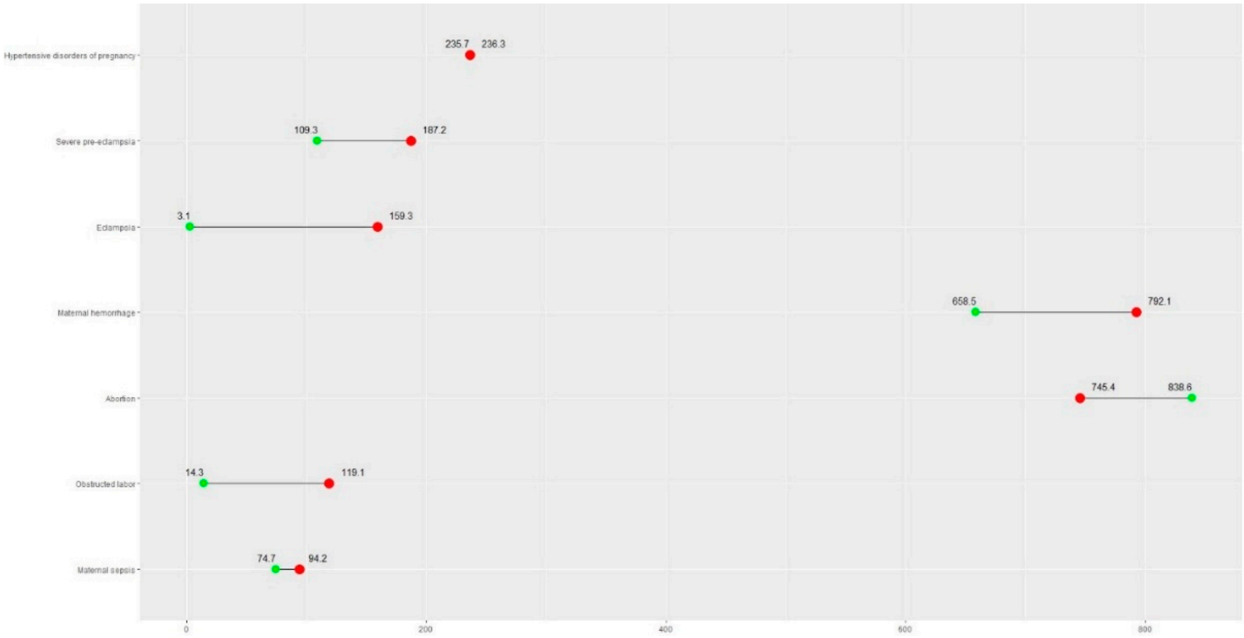

Figure S1. Change in Disability-Adjusted Life Years in Almaty from 2018 to 2020 by Maternal Disorder (per 100,000 population).

Figure S2 shows the direction of a change in DALY per 100,000 population from 2018 to 2020 by maternal disorder. There was a slight increase of 0.6 in the total DALY per 100,000 population for HDP from 2018 to 2020. Furthermore, there was an increase of 77.9 and 156.1 in the total DALY per 100,000 population for severe pre-eclampsia and eclampsia accordingly from 2018 to 2020.

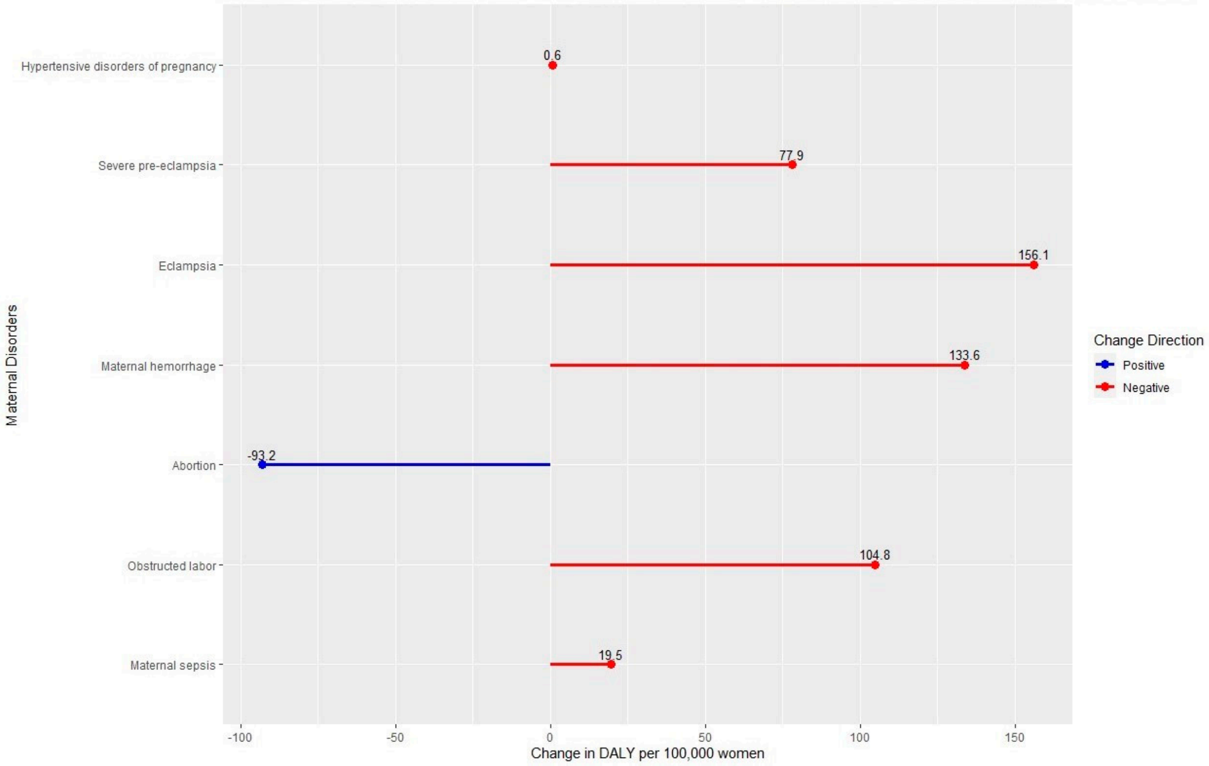

Figure S2. Change in Disability-Adjusted Life Years in Almaty from 2018 to 2020 by Maternal Disorder (per 100,000 population).
